# Cardiometabolic Risk Assessments by Body Mass Index *z*-Score or Waist-to-Height Ratio in a Multiethnic Sample of Sixth-Graders

**DOI:** 10.1155/2014/421658

**Published:** 2014-07-14

**Authors:** Henry S. Kahn, Laure El ghormli, Russell Jago, Gary D. Foster, Robert G. McMurray, John B. Buse, Diane D. Stadler, Roberto P. Treviño, Tom Baranowski, HEALTHY Study Group

**Affiliations:** ^1^Division of Diabetes Translation, Centers for Disease Control & Prevention, CDC Mail Stop F-73, 4770 Buford Highway, Atlanta, GA 30341, USA; ^2^The Biostatistics Center, George Washington University, Rockville, MD 20852, USA; ^3^School for Policy Studies, University of Bristol, Bristol BS8 1TZ, UK; ^4^Center for Obesity Research and Education, Temple University, Philadelphia, PA 19122, USA; ^5^Department of Exercise & Sport Science, University of North Carolina, Chapel Hill, NC 27599, USA; ^6^Division of Endocrinology, Department of Medicine, University of North Carolina School of Medicine, Chapel Hill, NC 27599, USA; ^7^Division of Health Promotion & Sports Medicine, Oregon Health & Science University, Portland, OR 97239, USA; ^8^Social & Health Research Center, San Antonio, TX 78210, USA; ^9^Children's Nutrition Research Center, Baylor College of Medicine, Houston, TX 77030, USA

## Abstract

Convention defines pediatric adiposity by the body mass index *z*-score (BMIz) referenced to normative growth charts. Waist-to-height ratio (WHtR) does not depend on sex-and-age references. In the HEALTHY Study enrollment sample, we compared BMIz with WHtR for ability to identify adverse cardiometabolic risk. Among 5,482 sixth-grade students from 42 middle schools, we estimated explanatory variations (*R* ^2^) and standardized beta coefficients of BMIz or WHtR for cardiometabolic risk factors: insulin resistance (HOMA-IR), lipids, blood pressures, and glucose. For each risk outcome variable, we prepared adjusted regression models for four subpopulations stratified by sex and high versus lower fatness. For HOMA-IR, *R* ^2^ attributed to BMIz or WHtR was 19%–28% among high-fatness and 8%–13% among lower-fatness students. *R* ^2^ for lipid variables was 4%–9% among high-fatness and 2%–7% among lower-fatness students. In the lower-fatness subpopulations, the standardized coefficients for total cholesterol/HDL cholesterol and triglycerides tended to be weaker for BMIz (0.13–0.20) than for WHtR (0.17–0.28). Among high-fatness students, BMIz and WHtR correlated with blood pressures for Hispanics and whites, but not black boys (systolic) or girls (systolic and diastolic). In 11-12 year olds, assessments by WHtR can provide cardiometabolic risk estimates similar to conventional BMIz without requiring reference to a normative growth chart.

## 1. Introduction 

The conventional definitions of pediatric adiposity depend on a measured body mass index (BMI, kg/m^2^) interpreted relative to a reference distribution (BMI normative growth charts) for sex and age [1–3]. Because it has body weight in its numerator, BMI reflects generalized (total-body) enlargement with a simplified correction (as height^2^) for skeletal size. Pediatric adiposity has been defined alternatively by abdominal size, most commonly a waist circumference, because increased truncal adipose tissue is correlated better than generalized adiposity with cardiometabolic dysfunction [4, 5]. Since waist circumference is a regional measurement, it reflects specifically only abdominal or central enlargement. It includes no correction for skeletal size. Thus, waist circumference also requires interpretation relative to its own reference distribution for sex and age. Comparisons of risk assessments in youth have generally found little difference between BMI and waist circumference in the ability of these indicators to identify cardiometabolic risk [6, 7].

The waist-to-height ratio (WHtR) is an adiposity indicator with waist circumference in the numerator and a simplified correction (as height) for skeletal size. WHtR does not depend on sex- or age-specific reference criteria [8–10]. In a large sample of US sixth-graders, we examined the performance of BMI *z*-score (BMIz, referenced to* CDC 2000* growth charts for the United States [11]) and WHtR for the purpose of cardiometabolic risk assessment.

## 2. Methods 

### 2.1. Study Design and Participants

Participants came from the baseline enrollment (sixth-grade students in 2006) of the HEALTHY Study, a cluster-randomized, controlled, primary prevention trial designed to improve indicators of adiposity and glycemic dysregulation among US middle-school children [12, 13]. Seven research centers recruited 42 middle schools with at least 50% of students eligible to participate in the federally subsidized National School Lunch Program or belonging to an ancestral minority group at increased risk of type 2 diabetes (Hispanic, non-Hispanic black, or Native American). A detailed protocol and background details about the HEALTHY Study are available for download at http://www.healthystudy.org/.

We restricted* a priori* our analytic sample to students with integer ages of 11 or 12 years who were able to participate in physical education and did not have known diabetes. From 5950 eligible enrollees, we excluded 13 students due to missing or invalid data for the adiposity indicators (height, weight, and waist circumference). Additional 239 students were excluded for lack of outcome cardiometabolic risk variables, and 216 were excluded for lack of information on pubarche (an adjustment variable associated with adrenarche, growth pattern, and insulin resistance) [14–16]. This left a final sample of 5482 participants with complete data.

Participant ancestry (Hispanic, non-Hispanic black, non-Hispanic white, other) and sex were self-reported. The study was approved by each research center's Institutional Review Board, and informed consent from parents and assent from students were obtained prior to data collection.

### 2.2. Adiposity Indicators, Blood Pressure, and Reporting of Pubarche

From measured height and weight, we calculated the sex- and age-specific BMIz based on the Centers for Disease Control and Prevention 2000 growth charts [11, 17]. Waist circumference was measured to the nearest 0.1 cm on bare skin just above the iliac crest following procedures of the National Health and Nutrition Examination Survey [18], and the WHtR was calculated.

Blood pressure was recorded three times using an Omron automated blood pressure monitor (with appropriate-size cuff) after the participant sat quietly for 5 minutes. The mean of the second and third recordings was used in all analyses. Pubarche was identified dichotomously from the participants' response to a standardized question on the appearance of underarm and pubic hair [19].

### 2.3. Laboratory Methods

Fasting blood samples were processed onsite and shipped to the HEALTHY central blood laboratory (Northwest Lipid Research Laboratories, University of Washington) [12]. Insulin was measured by an immunoenzymometric assay using a Tosoh 1800 autoanalyzer; the interassay and intra-assay precision analysis consistently showed a coefficient of variation (CV) < 10%. The assay had low cross-reactivity with human C-peptide (0%) and proinsulin (2%). Glucose analyses were performed on a Roche P module autoanalyzer using the hexokinase method. The homeostasis model assessment of insulin resistance (HOMA-IR) was calculated from glucose (mg/dL) and insulin (*μ*U/mL) concentrations using the following formula [20]:

(1) $$\begin{array}{c} \text{HOMA-IR}=\frac{\left(\text{glucose}*0.05551\right)*\left(\text{insulin}\right)}{22.5}. \end{array}$$

Measurements of total plasma cholesterol, cholesterol in the lipoprotein fractions, and triglycerides were performed enzymatically on the Roche Modular-P autoanalyzer using well-standardized methods. The interassay CVs were consistently <1.5% for total cholesterol and triglycerides and <2% for HDL cholesterol. We calculated the total-to-HDL cholesterol ratio (Tc/HDLc), a variable that strongly predicts cardiovascular disease in adults [21] and may be more strongly associated than LDL-cholesterol or HDL-cholesterol concentrations with pediatric adiposity [22, 23].

### 2.4. Statistical Analyses

In addition to stratification by sex, we chose* a priori *to stratify our analyses by “high fatness” or “lower fatness” because the ability of adiposity indicators to identify adipose tissue mass, ectopic fat, and cardiometabolic risk variables may be stronger among children in a higher fatness category [23–26]. As defined for this report, the high-fatness level included students who were above the sex-specific median value for both BMIz and WHtR; any student below the median for either adiposity indicator was designated lower fatness. For each fatness level, we prepared sex-specific, linear-regression models adjusted for ancestry (4 categories) and pubarche (yes/no) to estimate the associations of continuous adiposity indicators with the continuous cardiometabolic risk factors (outcomes). Since blood pressure varies with height in children [27, 28] our models for blood pressure outcomes included an additional adjustment for height which was entered as a continuous variable. For all variables except BMIz, we calculated descriptive statistics without using reference-based corrections for sex or age. For indicators or outcomes that departed markedly from a normal distribution (WHtR, HOMA-IR, Tc/HDLc, HDL cholesterol, triglycerides, and (only for lower-fatness students) diastolic blood pressure), we transformed the variable by log⁡_*e*_⁡ or inverse square root to approach normality prior to their use in regression models [29].

Our adjusted models estimated standardized beta coefficients (change in the outcome variable (in standard deviations) associated with change of one standard deviation in the adiposity indicator) for each cardiometabolic risk factor. PROC MIXED (SAS version 9.2; SAS Institute Inc., Cary, NC) was used to account for variability both within and between the school clusters. In these mixed models, the proportion of variation explained (*R* ^2^) by each adiposity indicator was calculated as the full model *R* ^2^ minus *R* ^2^ for a model omitting the adiposity indicator. To compare linear slopes among ancestral groups, mixed-regression models estimated nonstandardized, beta-regression coefficients; interactions were tested between each adiposity indicator and the three ancestries represented prominently in our sample (Hispanic, black, and white). We report as noteworthy those ancestral contrasts where *P* < 0.01.

## 3. Results

Characteristics of the analytic sample are presented by sex in Table 1. As expected for sixth-grade students [30], girls had greater height, insulin resistance, and triglycerides than boys. Following further stratification by fatness level, the distributions of adiposity indicators and ancestry are summarized in Table 2. Compared to the high-fatness groups in our sample, the lower-fatness groups had BMI and WHtR distributions that resembled more closely the general population of US youth in the same age range [31, 32].

Within the high-fatness subpopulations of either sex (Table 3), adiposity indicators explained 19%–28% of the variation in HOMA-IR, 4%–9% of the variation in circulating lipids (Tc/HDLc, HDL cholesterol, triglycerides), and 5%–9% of the variation in diastolic blood pressure. Adiposity indicators explained <3% of the variations in systolic blood pressure and fasting glucose. For each outcome variable in these high-fatness subpopulations, the effect sizes (standardized beta coefficients) associated with BMIz were similar to those associated with WHtR.

Within the lower-fatness subpopulations of either sex (Table 4), adiposity indicators explained 8%–13% of the variation in HOMA-IR and 2%–7% of the variation in circulating lipids. For Tc/HDLc and triglycerides, the standardized beta coefficients tended to be weaker for BMIz (0.13–0.20) than for WHtR (0.17–0.28). Adiposity indicators in these lower-fatness subpopulations explained <1% of the variations in systolic blood pressure, diastolic blood pressure, and fasting glucose.

A comparison between the two levels of fatness (Table 3 versus Table 4) demonstrates that for either adiposity indicator the associations with HOMA-IR were stronger among the high-fatness students (beta coefficients 0.43–0.52) than among the lower-fatness students (0.30–0.37). Similarly, both adiposity indicators were associated with diastolic blood pressure more strongly among high-fatness students (0.23–0.32; *P* < 0.001 for each of 4 beta coefficients) than among lower-fatness students (0.03–0.05; *P* > 0.05 for each of four beta coefficients). For identification of lipid outcomes, however, we found steeper beta coefficients only among high-fatness boys (compared to lower-fatness boys) whose adiposity was assessed by BMIz. For both sexes assessed by WHtR, the beta coefficients were similar across the fatness levels.

**Figure 1:**
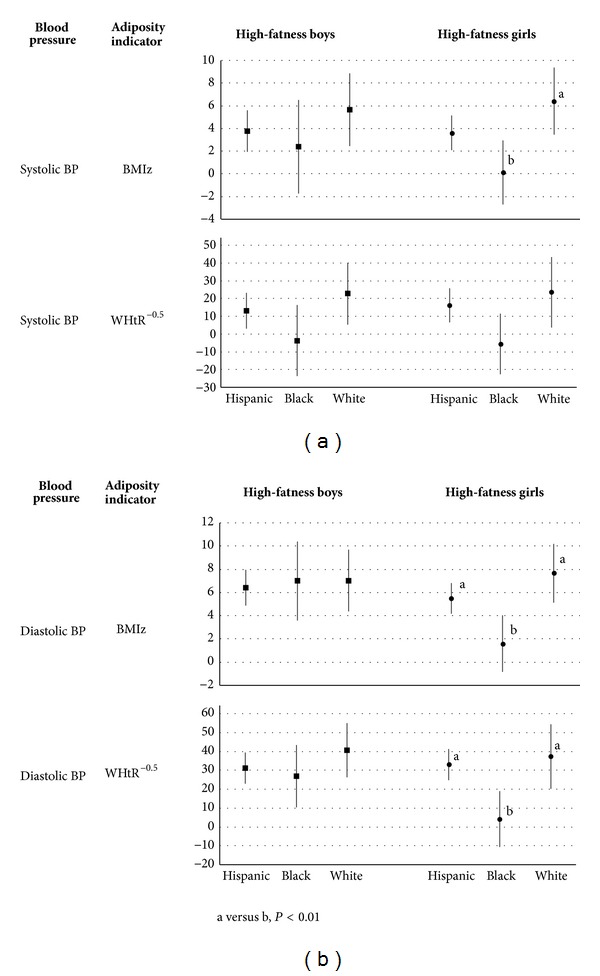
Within the high-fatness subpopulations, the point estimates (with 95% confidence intervals) represent here the slopes of the associations between blood pressure and either BMIz or WHtR for 3 predominant ancestral groups (Hispanic, black, and white). Black participants exhibited no linear associations between systolic blood pressure and increasing adiposity (a). Diastolic blood pressure was related to increasing adiposity for black boys, but not black girls (b).

The associations between adiposity indicators and risk variables were not notably different between the Hispanics, non-Hispanic blacks, and non-Hispanic whites except when related to blood pressure outcomes. Although adiposity explained 5%–7% of variation in diastolic blood pressure in the complete sample of high-fatness girls (Table 3), this relationship was extremely weak for high-fatness girls who were black, as indicated by slope point estimates close to zero (Figure 1). However, for high-fatness girls who were Hispanic or white, BMIz and WHtR had significant associations (*P* < 0.05) with diastolic pressure. Among high-fatness boys, we found no ancestral contrasts related to diastolic pressure.

Systolic blood pressure was not significantly associated with BMIz or WHtR for high-fatness blacks of either sex, but the association was present for high-fatness students who were Hispanic or white. An ancestral contrast (blacks compared to whites) related to systolic blood pressure was significant, however, only among the high-fatness girls assessed by BMIz (Figure 1; *P* < 0.01).

## 4. Discussion

In this large sample of middle-school students at increased risk of obesity and type 2 diabetes, we found that adiposity indicators BMIz (with reference to* CDC 2000* growth charts) and WHtR (without reference to sex and age) had similar utility for identifying adverse levels of cardiometabolic variables. Our findings are generally consistent with previous published reports, most of which were based on populations that had a wider age range or included less ancestral diversity. Earlier cross-sectional studies that compared continuous WHtR against BMI either without a normative growth reference [33–35] or with reference-based BMI *z*-scores/percentile ranks [23, 36] generally found that WHtR provided stronger associations with lipid outcomes, but BMI was superior for blood pressure outcomes. A recent report on sixth-grade students from Switzerland found that BMIz (referenced to* CDC 2000 *growth charts) and WHtR provided associations with blood pressures that were weak but nearly identical [37]. In studies of youth from the southern US, BMIz provided a slightly stronger association than WHtR with HOMA-IR [38] and fasting insulin [23], a relationship that was complicated by nonlinearity. Nationally representative, cross-sectional data from the US demonstrated that WHtR at ages 4–17 years was more strongly associated than BMIz with resting heart rate [36]. A longitudinal analysis from the same survey of adolescent and young-adult participants found that categorical WHtR predicted all-cause mortality before age 55 better than categorical BMIz (baseline ages 12–18 years) or BMI (ages 19–39) [39]. From the United Kingdom, a large study recently reported that WHtR and BMIz obtained at ages 7–9 years had similar associations with cross-sectional and prospective cardiometabolic risk factors in adolescence [40].

Given an approximately equal utility of BMIz and WHtR for pediatric health assessments, we should consider how these adiposity indicators might perform in different settings or in the future. BMIz values reported in the research literature depend on standardized protocols for measuring height and weight using calibrated, high-quality scales. In nonresearch settings, however, staff training and time pressures might not be so favorable to careful measurements [41]. The dependence of BMIz on normative growth references can be problematic because BMI-for-age reference values can yield discrepant inferences between populations, time periods [3], and ethnicities within a single country [42]. The International Obesity Task Force prepared a “worldwide” BMI growth reference based on large datasets from six countries [43], but subsequent reviews found that this international growth reference provided no advantage over national BMI growth references for the definition of excessive fat mass in youth [6] or prediction of subsequent cardiovascular risk in adulthood [44]. The World Health Organization (WHO) more recently developed a BMI-based growth reference [45], the utilization of which has been described as a cumbersome task in need of simplification [46]. Surveys from various clinical settings have found generally that the use of BMI-for-age reference values is suboptimal [47–50].

Advocates of the WHtR must address problems associated with the available protocols for measuring waist size. While tape measures are inexpensive and generally need little calibration, protocols for circumference measurement are still unfamiliar to many pediatric practitioners or clinic assistants. Our study carefully measured the waist circumference just above the iliac crest, an anatomic location endorsed by prominent researchers in the United States [18, 51] and Canada [52]. The WHO, however, recommends measuring waist circumference at the approximate midpoint between the lower margin of the last palpable rib and the top of the iliac crest [53]. Waist circumferences have been taken also at the level of the umbilicus, the “minimal waist,” and other sites [54, 55]. In an anthropometric study of diabetic youth, the iliac-crest protocol and WHO protocol demonstrated comparable reproducibility, but these alternative protocols yielded notable differences in the absolute value of a waist circumference obtained from the same participants [56]. A study of overweight youth found that the WHO waist-circumference protocol had a stronger association than the iliac-crest protocol with cardiometabolic risk [57], and studies of adult waist circumference have likewise suggested an advantage for the WHO protocol [58–60]. It follows that the WHtR values calculated from the iliac-crest and WHO protocols should not be casually substituted for each other. It is possible that if our HEALTHY Study had adopted the WHO instead of the iliac-crest protocol for its baseline anthropometry, the re-calculated WHtR indicator might have demonstrated stronger associations with cardiometabolic risk variables than those we report in this paper. However, such WHO measurements are not available in the HEALTHY Study.

Standardization of a single waist-circumference protocol would probably advance the widespread adoption of the WHtR as a low-cost adiposity indicator [55, 61]. As an alternative to the circumference, some pediatric investigators have described waist size in selected participants by measuring the external diameter sagittally (back-to-front) in the supine position [62–64]. Their reports suggest that standardization of a “sagittal abdominal diameter” protocol might further enhance studies that are cross-sectional or involve short-term follow-up of central adiposity, but this anthropometric method needs to be tested in larger datasets that represent general youth populations.

The physiological importance of tissues accumulated in the waist may help to explain why WHtR was more closely associated than BMIz with variations in the levels of circulating lipid markers among our lower-fatness participants (Table 4). An increase in waist size primarily marks expanding amounts of adipose tissue, including notably the visceral depot which is most strongly associated with an adverse metabolic phenotype [5, 65]. Variation in the waist circumference can explain more than 64% of the variance in the area [66] or volume [67] of visceral adipose tissue. An increase in BMI, by contrast, may substantially mark also the variations in gain of muscular weight or subcutaneous fat patterning that precede adulthood [1]. Along with the changes in fat mass, these variations in lean mass or superficial adipose tissue contribute to the BMI calculation while contributing relatively little to metabolic risk.

Although high-fatness Hispanic and white girls in our study demonstrated the expected associations between adiposity and blood pressure, we found among the high-fatness black girls (but not black boys) that neither BMIz nor WHtR had a significant association with blood pressure outcomes (Figure 1).   In comparison to young Hispanics and whites, young blacks tend to have abdominal adipose tissue relatively less in the visceral depot and more in the abdominal subcutaneous regions [68]. Another study has also reported that black girls' waist circumference around the same age was unrelated to their diastolic blood pressure [69]. Since both the BMIz and WHtR indicators demonstrated similar patterns of nonassociation with black girls' blood pressure, it may be that this absence of a correlation with blood pressure is due to factors operating primarily outside the abdominal region. Perhaps black girls benefit from an increased capacity to expand their lower-body (gluteofemoral), subcutaneous, adipose-tissue stores in a manner that would increase their total body weight yet protect them from cardiometabolic risk [70–73]. If this protective characteristic of black girls extends into their later years, it could help explain why adult black women in the US experience no increased cardiometabolic risk [74] or mortality [75] until their BMI reaches approximately 33 kg/m^2^.

## 5. Conclusions

If a well-standardized waist measurement comes into widespread use for clinical assessments of pediatric adiposity, patients and their families may improve their intuitive understanding of how excess adiposity contributes to adverse health risk. Pediatric health care providers, too, may find it more useful to recognize risk associated with a waist increment (corrected for height) than with a weight increment (corrected for height squared). Adoption of the WHtR could optimize both patient education and the tracking of risk [76]. Compared to BMI, the WHtR allows a simpler calculation without the necessity of squaring the child's height. Of interest to those concerned with child adiposity and cardiometabolic risk observed in different cultures or distinct time periods, the WHtR will facilitate comparisons based directly on anthropometric observations without using normative reference tables that may not be suitable to all populations [77–79].

## Figures and Tables

**Table 1:** Description of cross-sectional sample at grade 6 (*N* = 5,482).

	Boys (*n* = 2,585)			Girls (*n* = 2,897)		
	Median	Mean	SD	Median	Mean	SD
Continuous variables, units						
Age, years	11.8	11.8	0.5	11.7	11.7	0.4
Weight, kg	47.8	51.0	15.6	48.2	51.4	15.1
Height, cm	149.8	150.2	8.1	151.6	151.4	7.2
Body mass index, kg/m^2^	21.1	22.3	5.4	20.9	22.2	5.4
BMI *z*-score	1.118	0.958	1.090	0.917	0.827	1.075
Waist circumference, cm	72.5	75.8	15.3	72.9	75.5	14.0
WHtR	0.484	0.503	0.091	0.480	0.498	0.084
HOMA-IR	2.1	2.9	2.9	2.6	3.3	2.7
Total cholesterol/HDL cholesterol	3.0	3.1	0.9	2.9	3.1	0.8
HDL cholesterol, mmol/L	1.34	1.38	0.33	1.32	1.35	0.31
Triglycerides, mmol/L	0.84	0.99	0.59	0.87	1.01	0.57
Systolic BP, mmHg	107.5	108.1	10.2	106.5	106.8	9.7
Diastolic BP, mmHg	63.0	63.5	8.9	63.5	63.9	8.6
Glucose, mg/dL	94.0	94.4	6.6	92.0	92.6	6.6
Categorical variables, %		—			—	
Fatness level^a^ (high/lower)	45.9 / 54.1			44.6 / 55.4		
Ancestry^b^ (H/B/W/other)	52.2 / 18.4 / 21.2 / 8.2			53.9 / 19.7 / 18.0 / 8.4		
Pubarche (no/yes)	73.5 / 26.5			57.2 / 42.8		

^a^High-fatness students identified by being above the sex-specific median value for both BMI *z*-score and WHtR; the remaining students were designated as lower-fatness.

^b^H: Hispanic; B: non-Hispanic black; W: non-Hispanic white.

**Table 2:** Distributions of adiposity indicators and ancestral groups. The analytic sample is divided into four mutually exclusive subpopulations by sex and fatness level.

	Boys	Girls
High fatness^a^		
*n*	1,187	1,291
Body mass index, median (p25, p75)	25.9 (23.6, 29.2)	25.8 (23.4, 29.3)
BMI*z*, median (p25, p75)	1.91 (1.60, 2.20)	1.78 (1.41, 2.14)
WHtR, median (p25, p75)	0.574 (0.533, 0.625)	0.562 (0.524, 0.614)
Ancestry^b^ (H/B/W/other), %	61.0 / 13.1 / 18.8 / 7.2	58.6 / 18.4 / 15.4 / 7.5
Lower fatness		
*n*	1,398	1,606
Body mass index, median (p25, p75)	18.3 (16.9, 19.8)	18.3 (16.8, 19.9)
BMI*z*, median (p25, p75)	0.258 (−0.361, 0.754)	0.170 (−0.443, 0.657)
WHtR, median (p25, p75)	0.432 (0.408, 0.457)	0.436 (0.411, 0.460)
Ancestry^b^ (H/B/W/other), %	44.8 / 23.0 / 23.2 / 9.0	50.0 / 20.8 / 20.1 / 9.1

^a^High-fatness students identified by being above the sex-specific median value for both BMI *z*-score and WHtR; the remaining students were designated as lower-fatness.

^b^H: Hispanic; B: non-Hispanic black; W: non-Hispanic white.

p25 and p75 represent the 25th and 75th percentile values, respectively.

**Table 3:** Associations comparing standardized BMI *z*-score with standardized WHtR for estimating risk variable outcomes among high-fatness students (*N* = 2,478).

Risk outcome	Boys (*n* = 1,187)					Girls (*n* = 1,291)			
	BMI *z*-score		WHtR^−0.5^			BMI *z*-score		WHtR^−0.5^	
	Standardized beta	*R* ^2^	Standardized beta	*R* ^2^		Standardized beta	*R* ^2^	Standardized beta	*R* ^2^
	(95% CI)		(95% CI)			(95% CI)		(95% CI)	
Log HOMA-IR	0.52	0.277	0.48	0.234		0.47	0.213	0.43	0.195
	(0.48–0.57)		(0.43–0.53)			(0.42–0.52)		(0.38–0.48)	

Log Tchol/HDLchol	0.29	0.083	0.29	0.084		0.23	0.057	0.27	0.075
	(0.23–0.34)		(0.24–0.35)			(0.18–0.29)		(0.22–0.33)	

Log HDL cholesterol	−0.30	0.092	−0.28	0.077		−0.27	0.075	−0.28	0.079
	(−0.35–−0.24)		(−0.33–−0.22)			(−0.32–−0.21)		(−0.34–−0.23)	

Log triglycerides	0.24	0.061	0.24	0.058		0.19	0.037	0.22	0.052
	(0.19–0.29)		(0.18–0.29)			(0.14–0.24)		(0.17–0.27)	

Systolic BP^a^, mmHg	0.17	0.028	0.10	0.011		0.15	0.021	0.08∗	0.006
	(0.11–0.22)		(0.05–0.16)			(0.09–0.20)		(0.03–0.13)	

Diastolic BP^a^, mmHg	0.32	0.094	0.29	0.088		0.28	0.072	0.23	0.054
	(0.26–0.37)		(0.23–0.34)			(0.22–0.33)		(0.18–0.28)	

Glucose, mg/dL	0.04^†^	0.001	0.02^†^	0.000		0.08∗	0.005	0.06∗	0.003
	(−0.02–0.10)		(−0.04–0.07)			(0.03–0.13)		(0.01–0.12)	

^a^Models for blood pressures include an additional adjustment for height.

*R* ^2^ is the proportion of variation explained by the adiposity indicator.

All beta coefficients are *P* < 0.001 with exception of *(*P* < 0.05) and ^†^(*P* > 0.05), not significant.

Note that WHtR^−0.5^ is equivalent to 1/$\sqrt{\text{WHtR}}$ or 1/WHtR^0.5^.

**Table 4:** Associations comparing standardized BMI *z*-score with standardized WHtR for estimating risk variable outcomes among lower-fatness students (*N* = 3,004).

Risk outcome	Boys (*n* = 1,398)					Girls (*n* = 1,606)			
	BMI *z*-score		log WHtR			BMI *z*-score		Log WHtR	
	Standardized beta	*R* ^2^	Standardized beta	*R* ^2^		Standardized beta	*R* ^2^	Standardized beta	*R* ^2^
	(95% CI)		(95% CI)			(95% CI)		(95% CI)	
Log HOMA-IR	0.34	0.117	0.30	0.083		0.37	0.133	0.31	0.098
	(0.29–0.39)		(0.25–0.35)			(0.32–0.42)		(0.27–0.36)	

Log Tchol/HDLchol	0.18	0.031	0.26	0.063		0.20	0.041	0.28	0.074
	(0.13–0.22)		(0.21–0.32)			(0.15–0.25)		(0.23–0.33)	

Log HDL cholesterol	−0.19	0.038	−0.20	0.037		−0.22	0.049	−0.24	0.053
	(−0.24–−0.14)		(−0.26–−0.15)			(−0.27–−0.17)		(−0.29–−0.19)	

Log triglycerides	0.14	0.018	0.22	0.042		0.13	0.015	0.17	0.031
	(0.09–0.19)		(0.16–0.27)			(0.08–0.18)		(0.12–0.22)	

Systolic BP^a^, mmHg	0.03^†^	0.000	−0.01^†^	<0.001		0.04^†^	0.001	0.00^†^	<0.001
	(−0.02–0.08)		(−0.06–0.04)			(−0.01–0.09)		(−0.05–0.05)	

Log diastolic BP^a^	0.03^†^	0.000	0.05^†^	0.002		0.04^†^	0.001	0.05^†^	0.002
	(−0.03–0.08)		(−0.00–0.11)			(−0.01–0.09)		(−0.00–0.10)	

Glucose, mg/dL	0.06∗	0.004	0.05^†^	0.002		0.07∗	0.004	0.04^†^	0.002
	(0.01–0.11)		(−0.01–0.10)			(0.02–0.12)		(−0.02–0.09)	

^a^Models for blood pressures include an additional adjustment for height.

*R* ^2^ is the proportion of variation explained by the adiposity indicator.

All beta coefficients are *P* < 0.001 with exception of *(*P* < 0.05) and ^†^(*P* > 0.05), not significant.

## Abbreviations

BMIz: Body mass index *z*-score
BP: Blood pressure
CV: Coefficient of variation
CDC: Centers for Disease Control and Prevention
HDL: High-density lipoprotein
HOMA-IR: Homeostasis model assessment of insulin resistance
LDL: Low-density lipoprotein
Tc/HDLc: Total cholesterol/HDL cholesterol
WHtR: Waist-to-height ratio
WHO: World Health Organization.
